# Primary extrahepatic hydatid cyst of the soft tissue: a case report

**DOI:** 10.1186/1752-1947-6-404

**Published:** 2012-11-26

**Authors:** Salman Yousuf Guraya, Abdu Hassan Alzobydi, Shaista Salman Guraya

**Affiliations:** 1 Department of Surgery, College of Medicine Taibah University, Almadinah Almunawwarah, Saudi Arabia; 2 Department of Surgery, King Khalid Hospital, Najran, Saudi Arabia; 3 Department of Radiology College of Medicine, Taibah University, Almadinah Almunawwarah, Kingdom of Saudi Arabia

**Keywords:** Hydatid disease, *Echinococcus granulosis*, Soft tissue swelling, Surgical excision

## Abstract

**Introduction:**

Hydatid disease of the soft tissue is an exceedingly uncommon site to be affected by the tapeworm *Echinococcus*. The presentation is often vague and misleading. The diagnostic armamentarium has to be supplemented by a meticulously taken history and clinical examination.

**Case presentation:**

The present case report describes a 33-year-old Saudi male with a painless swelling in the right buttock which turned out to be a primary hydatid disease of the soft tissue. The lump was successfully excised surgically and the patient had an uneventful discharge.

**Conclusion:**

Surgical excision of the extrahepatic hydatid disease remains the mainstay of treatment; although medical treatment is available for the recurrent and disseminated disease.

## Introduction

Hydatid disease is a zoonotic infection caused by *Echinococcus*, a cestode of the Taeniidae family [1]. It is characterized by cystic lesions occurring in different parts of the human body. The disease is common in animal-raising regions and poses a significant public health problem in many areas worldwide. The disease is the most pathogenic zoonoses in the northern hemisphere with an annual incidence of 0.03 to 1.2 per 100,000 inhabitants [2]. Dairy farming seems to be a risk factor. Sixty percent of patients are involved in vocational or parttime farming, gardening, forestry, or hunting [3].

Liver is the most common organ involved (45-75%), followed by the lung (10-50%) [4]. The disease usually metastasizes to spleen, retroperitoneum, brain, bone, pancreas [5], and adrenal glands [6]. The current case report demonstrates hydatid disease in the soft tissue with a successful surgical cure.

## Case presentation

**Figure 1:**
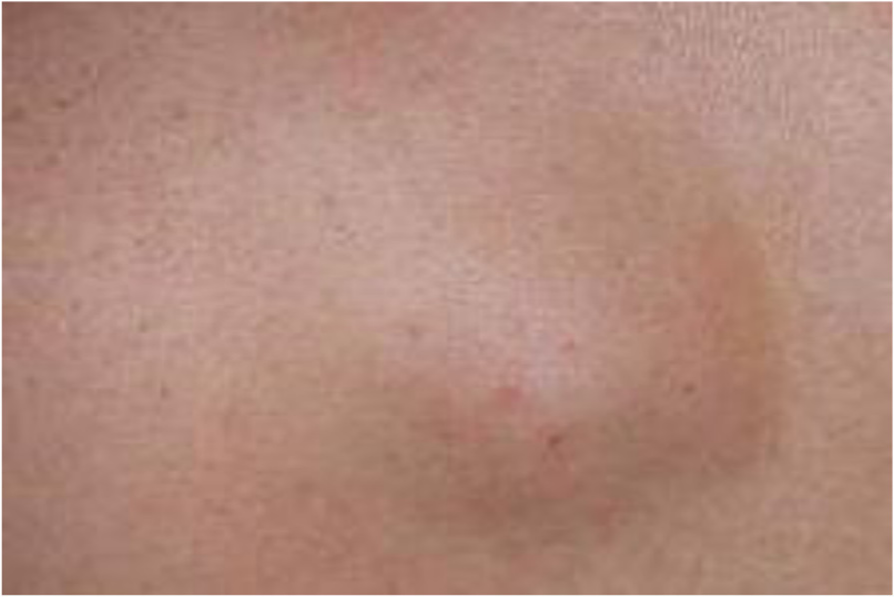
Preoperative view of the lump in the right buttock.

**Figure 2:**
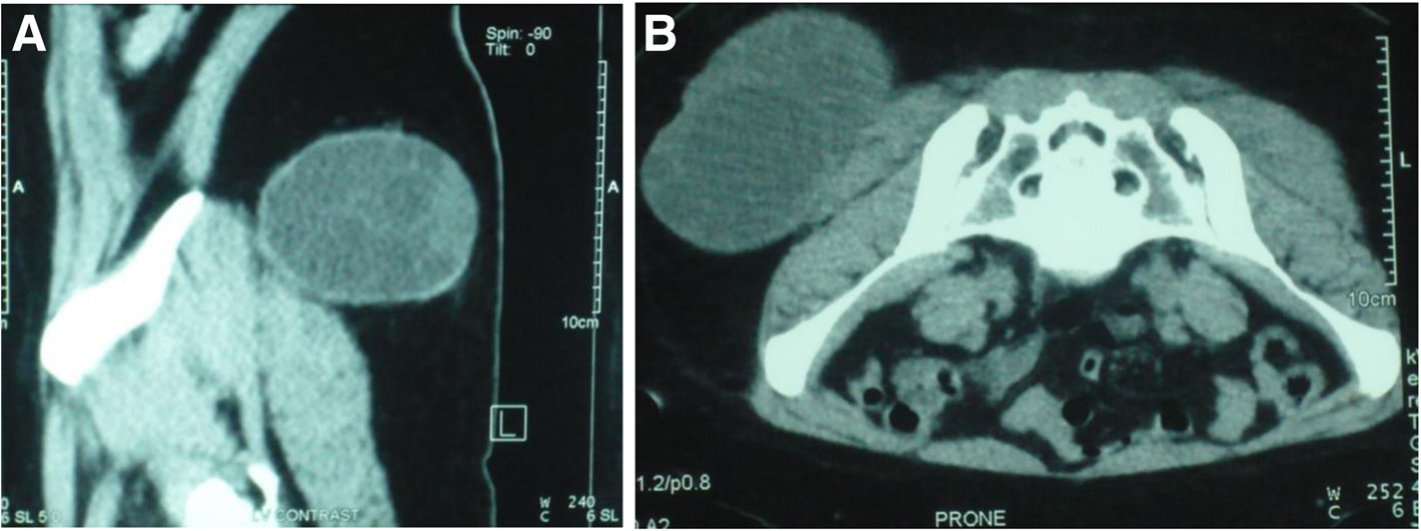
(A and B) Computed tomography; Parasagittal and axial view of the pelvis showing a cystic mass involving the right gluteus medius measuring 5x6cm with multiple daughter cysts inside the parent lesion.

**Figure 3:**
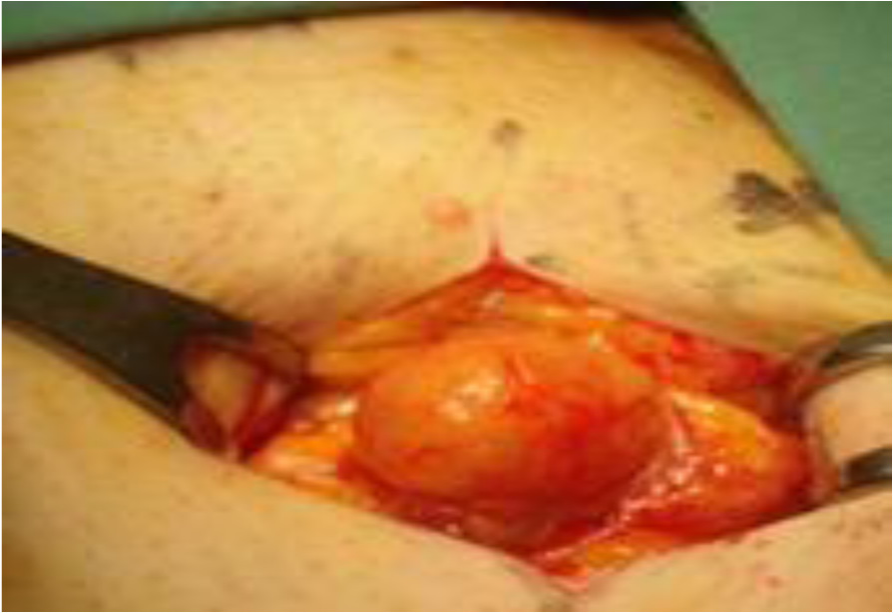
Per-operative view of the field showing the cystic mass being dissected.

**Figure 4:**
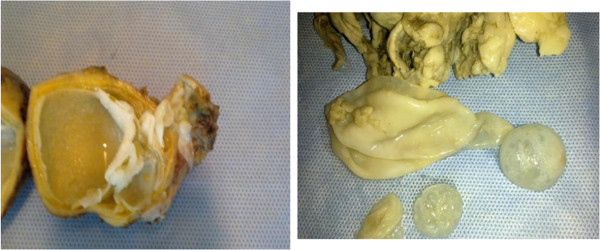
Cut section of the main hydatid cyst with daughter cysts.

**Figure 5:**
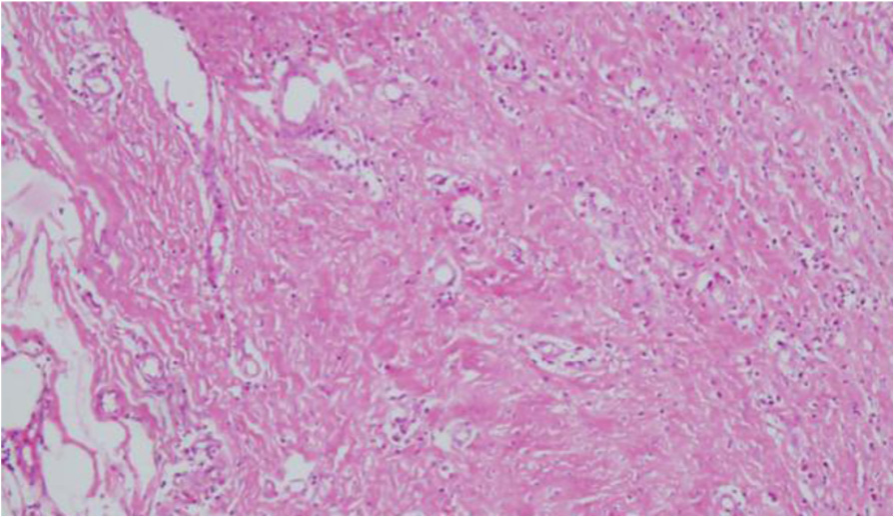
Histological view (hematoxylin and eosin stain X200) of the cyst showing thick fibrous wall and necrotic material.

A 33-year-old Saudi male presented to the surgical clinic of King Khalid Hospital Najran Saudi Arabia, with a slowly growing painless lump in the right buttock for about six months. The patient complained of fatigue and recurrent episodes of low grade fever. On examination, there was a soft and cystic, non-tender lump on the right buttock measuring about 4×4cm (Figure 1). The overlying skin was normal without any punctum or discharge. Except for eosinophilia in the complete blood count (CBC), the rest of baseline blood tests and chest X-ray were normal. The enzyme-linked immune-absorbent assay (ELISA) was positive for the *Echinococcal granulosis* antigens. Computed tomography (CT) scan of the concerned region showed an intact cyst with thin enhancing rim containing homogenous fluid contents (Figure 2 A and B). Later on, further inquiry from the patient confirmed his direct contact with the infected sheep a few months before. Surgical exploration of the mass was undertaken under general anesthesia. After appropriate packing of the surgical field with 20% hypertonic saline solution, the lump was completely excised (Figure 3). The lump was found to be a primary muscular hydatid cyst, attached to the right gluteus medius muscle, with multiple daughter cysts (Figure 4). The histopathology report detailed a circumscribed multilocular cystic lesion with a 2-mm thick fibrous wall (Figure 5). The cyst contained clear fluid with sand-like pasty material and calcified bodies. There were multiple daughter cysts with the same histological architecture. Following the surgical procedure, albendazole 10mg/kg/day was advised for 3 months to prevent recurrence. The patient had an uneventful recovery and was discharged home in a satisfactory condition.

## Discussion

Traditionally, hydatid disease, also called hydatidosis, has two clinical categories: cystic hydatidosis caused by *Echinococcus granulosus* and alveolar hydatidosis caused by *Echinococcus multilocularis*[7]. Infection begins with the ingestion of tapeworm eggs, which in the human intestine hatch into embryos that penetrate the small bowel mucosa, enter venules and travel via portal circulation to the liver. Hydatid cysts most often develop in the liver. However when embryos pass through this first filter, the second most frequent location is the lung. Hydatid cysts can occur anywhere in the body [8].

The symptoms and clinical signs depend on the structures and organs of the body affected by the pathogens. In hepatic affection, the symptoms include cholestatic jaundice, abdominal pain, fatigue, anaphylactic reaction, and weight loss [9]. Krasnigi et al. [7] reported primary hydatid disease of the gallbladder where the patient complained of vague upper abdominal pain and dyspepsia. While coughing, chest pain, and breathlessness are common presenting features of the pulmonary disease, Tekinbas et al. [10] successfully treated a patient of hydatid disease presenting with massive hemoptysis. Rupture of the pulmonary cyst is reported to occur in 34% to 64% of the patients [11]. Neil et al. [2] described a case of an 80-year-old man with primary extrahepatic alveolar Echinococcosis of the lumbar spine and the psoas muscle, presenting with recurrent episodes of low grade fever and low backache. The involvement of the bone and muscle is very rarely reported in the literature. A recent study has reported a total of 22 cases with soft tissue hydatid disease; all patients were from endemic areas and most from rural regions (90%) [12]. Most frequent locations were thigh (27%) and gluteal region (9%). Mean size was 2.5cm. Main symptom (70%) was painless, slow growing mass with normal overlying skin. Similarly, the present case report highlights a primary hydatid disease of the muscle presenting with a painless lump.

History of animal contact (especially dogs) and living in a sheep-raising or cattle-raising rural area is generally present. A plain radiograph of the of the region may show an unruptured cyst appearing as a spherical, well-circumscribed, homogenous opacity, particularly pronounced if calcified. Blood eosinophilia is reported to occur in 20% to 34% of patients [13]. It is a nonspecific sign because it may be seen in numerous other pathologies. A higher rate of eosinophilia was found in patients with ruptured cysts [14]. The serological tests including Casoni intradermal skin test, Weinberg complement fixation (CF) test, indirect hemagglutination (IHA) test, ELISA, and western blot (WB) are the frequently used laboratory tests for diagnosis of hydatid disease, with the reported sensitivity of 96.7%, 87.1%, and 100%, for IHA, ELISA, and WB, respectively [15]. Patients with a liver cyst are reported to have a higher rate of serologic test sensitivity than those having extrahepatic disease [16]. The serological tests are complementary to clinical and radiologic findings and can also be used in the follow-up of patients after surgical resection [17].

Ultrasonography (US) and CT have been reported to be the main diagnostic tools, with 85% and 100% sensitivity, respectively [18]. Although US is a reliable method for detecting echinococcal cysts, CT scans can define unique characteristics of hydatid cysts while also revealing additional small unsuspected lesions. If the cyst is intact, a CT scan with contrast enhancement may demonstrate a thin enhancing rim, calcifications, and daughter cysts. All these features were clearly demonstrated in the current case. In addition, an increase in the CT density of the affected organ and/or a thick wall should not negate a diagnosis of hydatid disease. On magnetic resonance imaging (MRI), cysts show low-signal intensity on T1- weighted images and high-signal intensity on T2- weighted images [19]. In general, MRI is not used in defining a lung hydatid cyst. However, fistulization of a hydatid cyst located on the liver dome through the transdiaphragmatic way to the pleural cavity can be visualized perfectly by MRI view.

Medical treatment with an antihelmenthic agent benzimidazole compound, either mebendazole or albendazole, is usually administered for the established hydatid disease. Albendazole is known to be more effective than mebendazole [20,21]. A standard dose of albendazole is 10 to 15mg/kg/d (taken twice daily). Owing to its hepatotoxicity, a 1-week to 2-week interval should be given between 3-week and 4-week cycles and treatment may last for 3 to 6 months. In some cases, praziquantel can be added to albendazole. There are reports that antihelmintic agents can reduce the size of cysts in some cases, however the results are not satisfactory and this treatment should be limited for disseminated and recurrent cysts or in cases where surgery is contraindicated [22]. Surgical excision of the cysts is the recommended treatment [23]. Per operatively, various scolicidal agents have been used including 0.04% chlorhexidine gluconate, 20% hyperomic saline, 0.5% silver nitrate, 10% povidone-iodine, and 2% formalin. The scolicidal activity of povidone-iodine was found to be better than that of hypertonic saline in experimental studies, but iodine has toxic effects on peritoneal mesothelial cells [24]. An aspiration technique has been proposed that employs an aspiration apparatus that can contain and remove the cyst fluid by suction, thereby minimizing the risk of peritoneal contamination [25]. The surgical excision of the soft tissue affection is markedly easy and straightforward than the visceral and deeply seated cysts, especially if perforated. Overall, the reported recurrence rates of hydatid cyst in the literature vary from 6.6% to 22% [26].

## Conclusion

To conclude, hydatid disease affects a wide range of organs and structures of the body including the soft tissue and muscle. The presentation is vague and misleading. A combination of carefully taken history, serological tests, and radiological imaging can clinch the diagnosis. The surgical therapy offers complete cure.

## Consent

Written informed consent was obtained from the patient for publication of this case report and accompanying images. A copy of the written consent is available for review by the Editor-in-Chief of this journal.

## Competing interests

The authors declare that they have no competing interests.

## Authors’ contributions

SYG conceived the idea, collected the data, and had a major contribution in writing the manuscript. SSG reported the images and improved the final layout. AHZ provided, analyzed and interpreted the patient data and helped in improving the final draft. All authors read and approved the final manuscript.
